# The Role of Internal Medicine or Hospitalist Co-Management in Surgical Specialties: Implications for Adult and Elderly Plastic Surgery Patients

**DOI:** 10.3390/medicina62030579

**Published:** 2026-03-19

**Authors:** Davide Quaglia, Elena Bocin, Massimo Robiony, Mario Alessandri Bonetti, Francesco De Francesco, Michele Riccio, Pier Camillo Parodi, Nicola Zingaretti

**Affiliations:** 1 Clinic of Plastic and Reconstructive Surgery, Academic Hospital of Udine, Department of Medicine (DMED), University of Udine, 33100 Udine, Italy; 2 Maxillofacial Surgery Department, Academic Hospital of Udine, Department of Medicine (DMED), University of Udine, 33100 Udine, Italy; 3 Department of Plastic and Reconstructive Surgery, University of Udine, 33100 Udine, Italy; 4 SODC Chirurgia Ricostruttiva e Chirurgia della Mano, AOU Ospedali Riuniti, 60126 Ancona, Italy

**Keywords:** internal medicine, microsurgical patient, plastic surgery, microsurgery, co-management, nutritional management, pre-habilitation, motor rehabilitation

## Abstract

*Background and Objectives*: Patients admitted to plastic surgery units increasingly present with multimorbidity, advanced age, diabetes, cardiovascular disease, chronic wounds, and complex metabolic requirements. In several surgical specialties, internal medicine specialist (IMS) co-management has been associated with improved clinical outcomes, yet its potential role in plastic surgery remains unexplored. *Materials and Methods*: A narrative scoping review conducted using systematic search principles was conducted using MEDLINE, Web of Science, Scopus, and Google Scholar from inception to 1 December 2025. Search terms combined “internal medicine,” “co-management,” and “surgery.” Studies assessing outcomes of IMS involvement in surgical inpatient care were included. Data on population, intervention characteristics, and outcomes were extracted and summarized. PRISMA recommendations were used to report the study selection process. The review focuses on adult and elderly surgical inpatients, as the available evidence is not applicable for pediatric populations. *Results*: Twenty-two articles met the inclusion criteria. IMS co-management demonstrated consistent benefits across multiple surgical specialties, including reduced length of stay, lower complication rates, improved metabolic and cardiovascular stabilization, enhanced perioperative optimization, and decreased costs. Despite the growing complexity of plastic surgery inpatients, no studies specifically evaluated IMS co-management in this field. *Conclusions*: Evidence from other surgical specialties suggests that structured IMS co-management may offer substantial benefits for plastic surgery patients, particularly those with multimorbidity, diabetes, severe burns, chronic wounds, or undergoing microsurgical reconstruction. Prospective studies are needed to determine its impact on patient outcomes, resource utilization, and clinical workflows within plastic surgery.

## 1. Introduction

Plastic surgery in modern clinical practice encompasses high-complexity procedures performed on patients with increasingly significant comorbidities. Elderly individuals requiring reconstructive surgery, patients with diabetes or peripheral vascular disease, individuals with polytrauma, and those with chronic or oncologic wounds frequently present with multimorbidity, polypharmacy, and substantial metabolic derangements. As a result, the demands of perioperative care have expanded beyond the traditional scope of surgical management.

In other surgical specialties—such as orthopedics, general surgery, vascular surgery, and oncology—co-management models involving internal medicine specialists (IMS) have demonstrated improvements in clinical outcomes, patient experience, and hospital efficiency. These models frequently reduce complications, optimize chronic disease management, shorten length of stay, and reduce readmission rates.

Despite the conceptual overlap between medically complex populations and plastic surgery inpatients, no systematic evaluation has addressed whether these benefits extend to plastic surgery. Understanding this potential role is increasingly important given the metabolic, cardiovascular, and systemic challenges observed in patients undergoing reconstructive or burn-related procedures.

This review aims to analyze evidence on IMS co-management in surgical specialties and explore its potential applicability to plastic surgery patients. Although plastic surgery frequently involves patient populations marked by advanced age, frailty, metabolic instability, and extended postoperative recovery, it remains largely underrepresented in the literature on perioperative internal medicine co-management. This constitutes a significant gap, given that the clinical complexity inherent to reconstructive, oncologic, and burn procedures is comparable to—and in some cases exceeds—that encountered in other surgical specialties where co-management has shown clear benefits. Bridging this gap is crucial to guide the development of future organizational models and to shape research priorities in plastic surgery [1,2,3,4].

## 2. Materials and Methods

This study was designed as a narrative (scoping) review conducted using systematic search principles, aimed at mapping and summarizing existing evidence on internal medicine or hospitalist co-management in surgical inpatients.

We searched the literature published between 2014 and 1 December 2025 using Medline (https://pubmed.ncbi.nlm.nih.gov (accessed on 1 December 2025)) and Reference Citation Analysis (https://www.referencecitationanalysis.com (accessed on 1 December 2025)). The database was searched using the keywords “Internal Medicine” OR “Surgical Patient” AND “Co-management”, returning 252 records. In the included literature, the term “co-management” encompassed heterogeneous models of collaboration between surgeons and internal medicine physicians, ranging from consultation-based models to more integrated approaches involving shared clinical responsibility, daily joint rounds, shared orders, or dedicated co-managed beds. These models were variably described across studies.

In addition to these, the reference sections for included studies were examined to harvest more papers for this review. Inclusion criteria were adult surgical inpatient studies assessing IMS or hospitalist involvement in perioperative or inpatient co-management study designs, including RCTs, cohort studies, case series, and articles reporting clinical or organizational outcomes. Exclusion criteria were outpatients and purely theoretical or editorial papers.

**Figure 1:**
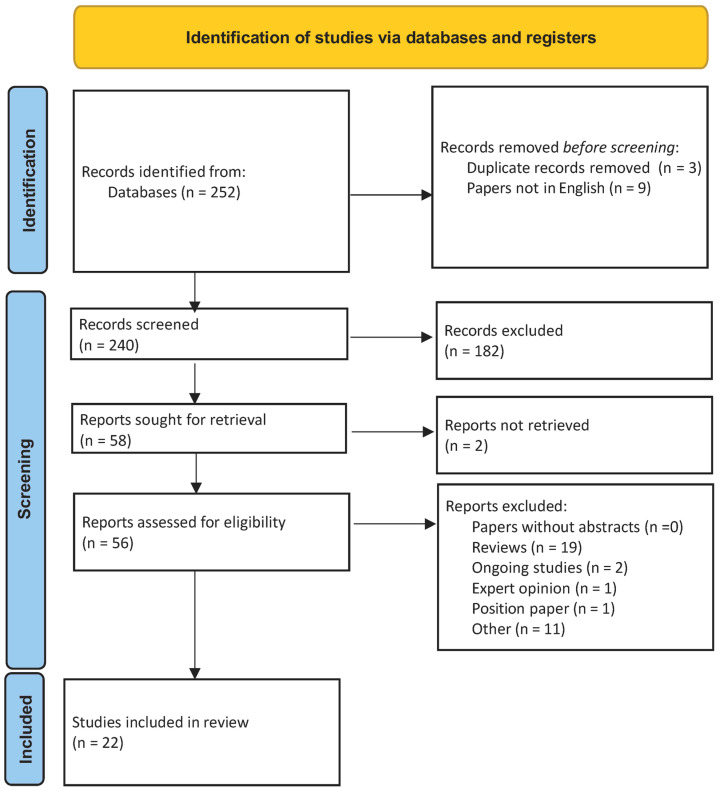
Preferred reporting items for systematic reviews and meta-analyses flow chart showing search criteria.

After removing duplicate papers and those not published in English (9), the eligibility criteria excluded papers without full text/abstracts, ongoing studies, expert opinions, position papers, and reviews (19). Either retrospective or prospective observational and experimental studies were included among the eligibility criteria. A total of 22 papers were used in this brief review. This manuscript is a narrative (scoping) review: PRISMA recommendations were used to transparently report the literature search and study selection process, but not to imply a formal systematic review (Figure 1); PRISMA checklist available at Supplementary Materials section.

Two authors (D.Q. and N.Z.) individually reviewed each article and rated them for importance and relevance to the topic. No formal risk-of-bias assessment was performed, given the heterogeneity of study designs and the narrative scoping nature of the review.

For each included article, data were extracted on surgical specialty, sample size, characteristics of the co-management model, and primary outcomes (LoS, complications, mortality, readmissions, and cost). Primary outcomes included clinical complications, length of stay, mortality, readmission rates, and cost-effectiveness. The reporting of included observational studies was guided by STROBE principles, with explicit identification of study design, population, and outcomes.

## 3. Results

No article concerning plastic surgery co-management by IMS was found; a total of 252 records were screened, and 22 concerning co-management by IMS in other surgical specialties were found.

Study designs were predominantly observational: retrospective cohort studies accounted for 55% (12/22), pre–post intervention studies for 32% (7/22), and prospective observational studies for 13% (3/22). The included studies were heterogeneous in design, population, and outcomes; therefore, results are presented narratively and grouped according to surgical specialty and primary clinical domains of interest; all included patients were adult and geriatric surgical inpatients.

Papers selected and outcomes extracted are summarized in Table 1; on the other hand, Table 2 summarizes the distribution of surgical specialties across the included studies.

A reduction in length of hospital stay (LoS) was reported in 68% of studies (15/22), particularly in orthopedic, vascular, neurosurgical, and anesthesiology-led settings. Lower rates of medical or overall complications were observed in 73% of studies (16/22), including reductions in delirium, cardiovascular events, infections, and pain-related outcomes.

A significant reduction in short-term or in-hospital mortality was documented in 32% of studies (7/22), most consistently in vascular, oncologic, cardiac, and frail geriatric surgical populations, while the remaining studies reported no increase in mortality.

Time to surgery (TTS) was specifically assessed in orthopedic hip-fracture cohorts and showed a significant reduction in 100% of studies evaluating this outcome (3/3). Improvements in process-of-care indicators—such as adherence to deep vein thrombosis prophylaxis, initiation of osteoporosis treatment, glycemic control, and pain management—were reported in 77% of studies (17/22).

Reductions in ICU or medical ward transfers were observed in 55% of studies (12/22), and 30-day readmission rates were reduced or unchanged in 82% of studies (18/22), with no study reporting an increase attributable to co-management.

## 4. Discussion

In this manuscript, the term “internal medicine specialist” refers to physicians with a coordinating and integrative role in the perioperative management of medically complex surgical patients, often working in close collaboration with other specialists such as psychiatrists, rehabilitation physicians, nutritionists, and intensivists.

### 4.1. Increasing Medical Complexity in Modern Surgical Populations

**Figure 2:**
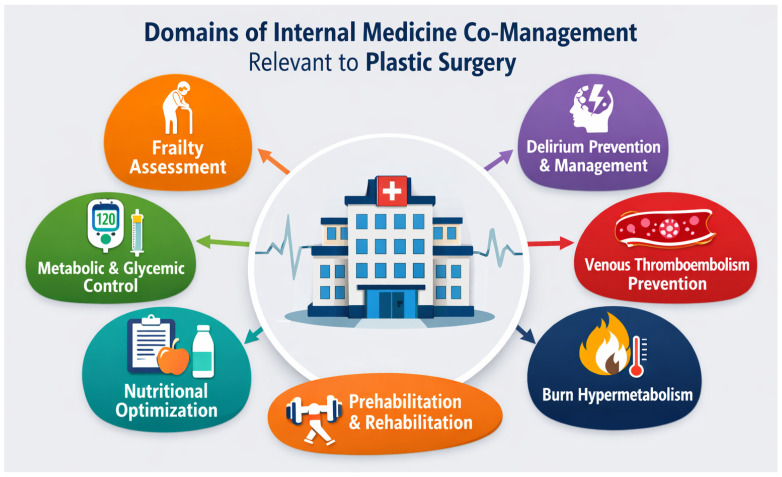
Domains of internal medicine co-management relevant to plastic surgery.

Advancements in surgical techniques have enabled progressively more medically complex patients—including older, frail individuals and those with multiple comorbidities—to become candidates for surgical interventions. This evolution has necessitated greater involvement of internal medicine (IM) physicians throughout the perioperative course. IM physicians increasingly assume a proactive and coordinated role in preoperative optimization of comorbidities, postoperative complication management, and facilitation of functional recovery. Such involvement is structured either through consultative models or formal co-management arrangements integrated into routine perioperative care. To date, however, the scientific literature lacks studies specifically addressing co-management within plastic surgery wards. Figure 2 summarizes the main clinical domains in which internal medicine co-management may contribute to perioperative optimization and overall patient management in plastic surgery settings.

### 4.2. Orthopedic Surgery as a Precedent for Co-Management Models

Orthopedic surgery was among the first specialties to adopt this organizational approach, leading to the development of orthogeriatrics, which integrates geriatricians into orthopedic teams caring for elderly patients with fractures. While this proactive involvement of IM was frequently associated with favorable clinical and organizational outcomes across multiple surgical specialties, it also introduces additional organizational complexity and costs, including increased diagnostic investigations and expanded multidisciplinary team participation involving nursing, physiotherapy, social work, and occupational therapy. One of the earliest and most successful examples of surgeon–internist integration has been the management of hip fracture patients [27]. Hip fracture is considered a deferrable emergency, as outcomes are strongly influenced by surgical timing, yet it predominantly affects highly frail patients and is therefore associated with elevated perioperative mortality.

The integration of internist or geriatrician expertise with orthopedic surgery may assume various organizational configurations, including on-call consultation, scheduled daily medical presence within orthopedic wards, or allocation of jointly managed beds. Across these models, 30-day mortality has consistently been lower than with standard care, with reductions proportional to the degree of interdisciplinary integration [27,28]. Roberts AJ et al. demonstrated, in a cohort of 517 patients treated for hip pathology, a significant reduction in time from admission to surgery and overall hospital length of stay among patients managed through internal medicine or hospitalist co-management; although the proportion of procedures performed under spinal anesthesia increased, no statistically significant differences were observed in readmission or mortality at 30 days, 90 days, or one year [13]. Similarly, Tsunemitsu A et al., in a single-center retrospective cohort study, reported reductions in in-hospital mortality, improvements in patient safety and pain control, and enhanced perceptions of care quality and resident education following implementation of co-management [17].

### 4.3. Multidisciplinary Integration and Extension of Orthogeriatric Models

A defining feature of the orthogeriatric model is structured multidisciplinary and multiprofessional integration, enabling coordinated care delivery by orthopedic surgeons, geriatricians, nurses, physiotherapists, and allied health professionals [29,30]. Initially developed for hip fracture management, this model has subsequently been extended to polytrauma, pelvic fractures, and spinal surgery. Incorporation of multidimensional geriatric assessment within orthopedic practice has proven effective even in more complex clinical contexts. Accordingly, orthogeriatric co-management has expanded beyond hip surgery alone.

Fitzgerald SJ et al. reported statistically significant improvements in mean hospital length of stay for total knee replacement, increased rates of discharge home following total knee replacement and primary total hip arthroplasty, reduced complication rates, and lower 30-day readmission rates in comanaged patients [19]. Moreover, orthogeriatric co-management may exert favorable effects on long-term functional outcomes and the risk of subsequent fractures [26].

### 4.4. Postoperative Delirium as a Target of Co-Management

Orthogeriatric co-management in patients undergoing orthopedic procedures for native or periprosthetic joint infections and nontraumatic surgery has demonstrated positive effects on the recognition of medical complications, including delirium [20]. Consistently, the odds of developing subsyndromal delirium or overt delirium have been shown to be lower in orthogeriatric cohorts [14]. Delirium represents a frequent postoperative complication in elderly surgical patients and is associated with prolonged hospitalization, cognitive impairment, and increased short-term mortality [31]. Reported prevalence ranges from 10% to 70%, depending on diagnostic criteria, patient characteristics, and surgical procedures, with particularly high rates following cardiac and orthopedic surgery [32,33].

Effective delirium management relies on the identification of high-risk patients, early diagnosis, and timely intervention. Delirium is characterized as an acute confusional state with fluctuating symptoms representing an acute or subacute deviation from baseline mental status [31]. Postoperative delirium has also been reported following head and neck reconstruction [34], oral reconstruction [35], and dermatologic surgery [36]. According to DSM-5 criteria, delirium involves acute disturbances in attention, awareness, and cognition with a fluctuating course [37]. Diagnostic tools include the Confusion Assessment Method and the Delirium Symptom Interview [38,39]. While delirium management often requires psychiatric expertise, early recognition, risk stratification, and correction of medical triggers represent key domains of internal medicine involvement.

Pathophysiological mechanisms proposed include oxidative stress [40] and neuroinflammation [41]. Management strategies encompass both pharmacological and non-pharmacological approaches, such as judicious use of haloperidol or ketamine in selected cases, patient reorientation, early mobilization, and early initiation of rehabilitation [38]. Electrolyte imbalances and nutritional deficiencies constitute important predisposing factors, and their correction represents a key domain of internist involvement. Given that plastic surgery departments routinely manage elderly patients, these considerations suggest a potential role for hospitalist participation [34,35,36].

### 4.5. Thromboembolic Risk in Plastic and Reconstructive Surgery

As in orthopedic populations, prevention of thromboembolic events and maintenance of hemorrhagic balance are fundamental components of patient safety. In microsurgery and complex reconstructive procedures, the risk of venous thromboembolism (VTE) is increased; in particular, autologous breast reconstruction is associated with nearly double the VTE risk compared with mastectomy or lumpectomy alone. Identified risk factors include advanced age, prior VTE, and malignancy, with immediate reconstruction conferring additional risk.

Prophylactic strategies combining low molecular weight heparin, mechanical compression devices, and early mobilization have demonstrated effectiveness, particularly when guided by the modified Caprini risk assessment model. However, low molecular weight heparin has been associated with increased hemorrhagic complications compared with unfractionated heparin. Patients with polypharmacy and multiple comorbidities undergoing complex microsurgical reconstruction, therefore, require a structured clinical framework, within which co-management may play a central role. Similar considerations extend to long-term immobilized patients, including those with extensive burns or complex lower-limb reconstructions [42].

### 4.6. Metabolic Dysregulation and Wound Healing

Surgical stress universally induces metabolic disturbances, including hypercatabolism, insulin resistance, and hyperglycemia, rendering metabolic control a critical perioperative issue across specialties, including plastic surgery [43]. Diabetes-related autonomic neuropathy can further complicate perioperative management and is independently associated with increased mortality [44,45]. Current guidelines recommend individualized antiplatelet management and perioperative glycemic targets between 80 and 180 mg/dL, with frequent glucose monitoring [45,46,47]. These metabolic alterations directly impact wound healing, which represents a central outcome in plastic and reconstructive surgery; in fact, the skin, as the principal organ treated in plastic surgery, plays a central role in homeostasis and wound healing. Diabetes, hypertension, and inflammatory conditions impair wound repair, with diabetic wounds affecting 10–25% of diabetic patients and characterized by delayed healing due to microvascular dysfunction, neuropathy, and chronic inflammation [44,45]. In breast cancer surgery, the interaction between type 2 diabetes and comorbidities significantly increases postoperative complication risk, accounting for up to 42% of adverse outcomes [48].

### 4.7. Nutritional Management and Burn Care

Current surgical guidelines restrict perioperative nutritional support to high-risk patients, aiming to preserve protein balance and immune function. Inadequate nutritional management is associated with increased morbidity, prolonged hospitalization, and higher mortality [49]. Internist-led nutritional assessment and support are particularly relevant in burn patients, who experience a severe and prolonged hypermetabolic response requiring early enteral feeding, careful caloric balance, and adjunctive therapies to preserve lean body mass and immune competence [50]. Burn care represents a highly specialized setting and is mentioned here only as an example of extreme medical complexity; however, its comprehensive management goes beyond the scope of this review.

### 4.8. Prehabilitation and Rehabilitation Pathways

Another relevant domain in which surgical patient co-management could play a role is pre-habilitation; we define pre-habilitation as the rehabilitation that occurs before surgery to help optimize a patient’s physical strength and general balance. “Pre-hab” strategies (general and targeted conditioning exercises, nutritional interventions, psychological well-being, and smoking cessation) have been successful in autologous breast microsurgical reconstruction [51]. Studies have shown that in breast reconstruction patients, pre-habilitation was beneficial in shoulder range of motion and upper extremity functional recovery, in post-operative pain reduction, and in increasing chances of feeling recovered sooner after surgery [42,52]. More generally, pre-rehabilitation in breast cancer patients improved outcomes, including physical function, quality of life, and psychosocial variables, with a preference for multimodal pre-habilitation compared to a unimodal one [53]. Investigating other surgical fields, “pre-hab” strategies are associated with some degree of benefit in upper gastrointestinal tract surgery [54], general abdominal surgery [55], head and neck oncological surgery [56], gynecological oncological surgery [57], and larynx cancer surgery [58]; nevertheless, further studies are needed to investigate pre-habilitation impact on specific outcomes such as the oncological outcome itself or the procedure complication rate.

On the other hand, co-management could be implemented in rehabilitation strategies; literature is consistent in terms of reduction in length of hospital stay and early recovery after surgery for specific motor impairment and after severe burn medical and surgical management. In burn survivors, rehabilitation plays a key role in terms of preventing scar contraction and further surgical intervention and restoring lean body mass, glucose and protein metabolism, cardiorespiratory fitness, and muscle strength [25,59]. As for plastic surgery patients, the hospitalist assessment and intervention could play a role in lymphedema and venous drainage management in post-surgical/post-oncological complications, burn scarring treatment, and chronic non-healing ulcers [59,60,61,62,63], even though no strong evidence has been reported so far. As for lymphedema, a truly integrated multidisciplinary approach is essential for treatment. Combining microsurgical techniques with reductive procedures should be recognized as an effective strategy, while rehabilitation must be central to treatment, not only for patients who are unsuitable for surgery but also as part of pre- and post-operative care. As such, the involvement of specialized plastic reconstructive surgeons and physical and rehabilitative medicine physicians is crucial to meet patients’ needs and enhance outcomes. In the management of upper limb lymphedema, for instance, instrumental physical therapy has been shown to reduce lymphedema volume and regulate chronic inflammation; extracorporeal shockwave therapy combined with complete decongestive therapy significantly improved volume, extracellular water ratio, and skin thickness in patients with breast cancer-related lymphedema, thus being effective in lymphedema treatment. Intermittent pneumatic compression has also shown promise with reported improvements in limb circumference and tissue tonicity over a 3-year period, although optimal pressure levels remain debated.

Physical activity, widely supported for cancer patients, is recommended for lymphedema management due to its benefits on overall survival, physical function, and quality of life. Despite the recognized advantages, barriers such as fatigue, competing responsibilities, and environmental factors often limit exercise adherence. Exercise therapy is also crucial for lower limb lymphedema, commonly combined with compression therapy. Brief exercise with compression bandages significantly reduced limb volume and tissue stiffness. However, there remains no consensus on the optimal exercise prescription for lymphedema patients, though physical activity should be strongly encouraged as part of comprehensive cancer-related lymphedema management due to its documented positive effects [60].

### 4.9. Evidence from Other Surgical Specialties and Patient Selection

The role of IM specialists in surgical co-management is well established in neurosurgery [21,23,59,63,64,65,66,67,68,69,70,71], maxillofacial surgery [72,73], vascular surgery [9,10,12,16,74], urology [7,24], oncologic surgery [8,11,18], cardiac surgery [22], and ENT surgery [6]. Across these settings, co-management has been associated with reductions in surgical delays, shorter hospital stays, and fewer minor complications. Nonetheless, a clear definition of roles and responsibilities remains essential to prevent ambiguity and workload imbalance [21,66,67,68,69,70,71].

Frailty stratification using validated multidimensional assessment tools is recommended to identify patients most likely to benefit from co-management and to guide individualized perioperative care planning; despite diagnosing and stratifying frailty in the geriatric population poses significant challenges, appropriate scales as tools that are both effective and appropriate for specific care settings have been implemented: the multidimensional assessment (MDA) is a fundamental skill for geriatricians and internists, involving a multidisciplinary and multiprofessional diagnostic process that evaluates the patient’s functional status to implement strategies aimed at preventing deterioration and reducing frailty. According to frailty stratification, not all patients could be candidates for co-management by IMS in terms of mortality and complication rates, which demonstrated a significant decrease [5,15,75,76,77].

## 5. Proposed Clinical Framework for Internal Medicine Co-Management in Plastic Surgery

Although plastic surgery-specific investigations were not identified within the available literature, the escalating medical complexity of patients undergoing reconstructive procedures substantiates the necessity for a structured co-management paradigm. Drawing upon evidence extrapolated from other surgical disciplines and recurrent clinical circumstances encountered in plastic surgery, we propose a pragmatic framework for involvement of the internal medicine specialist (IMS) tailored to this domain.

Plastic surgery increasingly treats elderly, multimorbid individuals affected by diabetes mellitus, cardiovascular disease, chronic kidney disease, malnutrition, and frailty. Microsurgical reconstruction, chronic wound care, oncologic reconstruction, and burn management frequently demand meticulous perioperative metabolic regulation, optimization of comorbid conditions, and prevention of systemic complications. Within this context, IMS participation should not be interpreted as a replacement for surgical decision-making but rather as a complementary and coordinating function focused on perioperative medical optimization.

### 5.1. A Tiered Model of Co-Management May Be Considered

1. **Consultative Model**—The IMS provides targeted consultation upon request for specific medical concerns (e.g., uncontrolled diabetes, exacerbation of heart failure, and anticoagulation management).
2. **Shared-Care Model**—The IMS and plastic surgeons conduct joint rounds in high-risk patients, sharing responsibility for medical management and pharmacologic adjustments.
3. **Integrated Perioperative Model**—IMS involvement commences preoperatively, including risk stratification, medication reconciliation, and metabolic optimization, and continues through postoperative monitoring and discharge planning.

Selection of patients for higher-intensity models may include advanced age, suboptimal glycemic control, multiple comorbidities, frailty, or anticipated prolonged hospitalization.

### 5.2. Practical Clinical Scenarios in Plastic Surgery

To illustrate potential applications of co-management in plastic surgery, several representative clinical situations may be considered:

**Microsurgical Reconstruction in Diabetic Patients:**

Free flap surgery necessitates strict glycemic regulation to minimize infection, flap thrombosis, and impaired wound healing. IMS co-management may facilitate preoperative HbA1c optimization, control of perioperative glucose variability, electrolyte monitoring, and individualized venous thromboembolism (VTE) prophylaxis.

**Chronic Wound Reconstruction in Elderly Frail Patients:**

Patients undergoing reconstruction for pressure injuries or chronic ulcers frequently present with sarcopenia, malnutrition, anemia, and polypharmacy. IMS participation may support nutritional evaluation, correction of metabolic derangements, delirium-prevention strategies, and comprehensive medication review.

**Postoperative Metabolic or Hemodynamic Instability:**

Complex reconstructive procedures may be complicated by fluid imbalance, electrolyte disorders, acute kidney injury, or cardiac decompensation. Early IMS involvement may enable prompt recognition and coordinated management, potentially reducing complications and hospital length of stay.

### 5.3. Organizational and Implementation Considerations

For effective implementation, institutions introducing IMS co-management within plastic surgery should define:

- Explicit criteria for patient referral or automatic activation of co-management
- Clearly delineated responsibilities regarding prescribing authority and shared orders
- Structured communication pathways between teams
- Outcome-monitoring systems, including complication rates, readmissions, and duration of hospitalization

Such frameworks may facilitate the transition from ad hoc consultation toward structured interdisciplinary collaboration.

## 6. Limitations and Future Directions

This review does not aim to demonstrate the effectiveness of internal medicine co-management in plastic surgery but rather to explore its biological and organizational plausibility based on evidence from other surgical specialties; some limitations warrant consideration. First, the literature specifically addressing co-management within plastic surgery is scarce, requiring extrapolation from other surgical specialties. Second, most available studies are observational or pre–post in design, limiting causal inference. Third, publication bias toward positive co-management outcomes cannot be excluded. Finally, variability in organizational models and outcome measures across studies limits generalizability. These limitations underscore the need for dedicated prospective and comparative studies in plastic surgery. The review focuses primarily on the coordinating role of internal medicine within multidisciplinary surgical care and does not aim to replace the expertise of other medical specialties involved in perioperative management.

It should be emphasized that the potential role of internal medicine co-management in plastic surgery is inferred by extrapolation from other surgical specialties and remains hypothetical, as no direct evidence in plastic surgery settings is currently available. It is important to note that outcomes associated with internal medicine involvement may differ substantially according to the intensity of the co-management model. More integrated models, characterized by shared clinical responsibility and structured daily involvement, appear more likely to impact clinical and organizational outcomes than consultation-only approaches. Future investigations should focus on plastic surgery subspecialties such as breast and cutaneous oncology, microsurgical reconstruction, and burn care, evaluating both patient-centered outcomes and system-level measures.

## 7. Conclusions

Although direct evidence in plastic surgery is lacking, data from other surgical specialties suggest that internal medicine co-management may represent a promising organizational model for selected plastic surgery patients. These considerations should be interpreted with caution and warrant dedicated prospective studies. Populations such as the elderly, burn patients, those undergoing microsurgical reconstruction, and individuals with diabetes or chronic wounds represent ideal candidates for IMS involvement.

Future research should focus on prospective evaluations of co-management models within plastic surgery to quantify their impact on outcomes such as complications, length of stay, readmissions, and cost-effectiveness. Establishing structured collaborative pathways may significantly enhance the quality, safety, and efficiency of care in plastic surgery wards.

## Figures and Tables

**Table 1:** Studies included in the review process.

Authors	Year	St	N. of Patients	Specialty	Outcome	Results
Montero Ruiz E et al. [5]	2014	Retrospective Observational Study	1629	ENT	Mortality, hospital stay length, and costs	Reduced LoS and costs
Fernández-de-Velasco D et al. [6]	2022	Observational retrospective cohort study	91	Cardiac Surgery (Post-sternotomy mediastinitis treatment with multidisciplinary management)	Impact of the co-management model of care vs. the standard model in patients diagnosed with PSM: survival time and treatment failure rate	A co-management care model reduced overall mortality in patients diagnosed with post-sternotomy mediastinitis
Giannotti C et al. [7]	2022	Single-center, nonrandomized, before-and-after study,	207 (107 control group; 90 co-management group)	Urology		Significant reduction in grade I-V complications and in 1-year readmissions in the co-management group; no difference in terms of 1-year mortality
Shahrokni A et al. [8]	2020	Retrospective cohort study	1892 undergoing surgical treatment for cancer (872 control group; 1020 co-management group)	Oncologic Surgery	90-day mortality; adverse surgical outcomes	Geriatric comanaged patients were associated with significantly lower 90-day postoperative mortality; adverse surgical events were not significantly different between groups
Thillainadesan J et al. [9]	2022	Monocentric pre-post study co-management intervention	302 (150 patients in the preintervention group and 152 patients in the postintervention group)	Vascular Surgery	Hospital-acquired geriatric syndromes, delirium, and LoS	Significant reductions in hospital-acquired geriatric syndromes (delirium and cardiac and infective complications) after implementing geriatric co-management; LoS unchanged
Iberti C T et al. [10]	2016	Monocentric pre-post study		Vascular Surgery	In-hospital mortality, length of stay, 30-day readmission rate, pain scores, and patient safety metrics	After two years of implementation, the co-management service reduced complications, mortality, and pain scores among high-risk vascular surgery patients
McMillan S [11]	2021	Monocentric observational	1687 (931 co-managed patients)	Oncologic Surgery	Mortality	Geriatric co-management was associated with lower 90-day postoperative mortality regardless of the degree of frailty
Mudge AM [12]	2020	Monocentric prospective pre-post intervention	235 surgical patients (112 pre-intervention 123 post-intervention)	Vascular Surgery	Primary: LoS, delirium, and functional decline. Secondary: medical complications and discharge destination.	In the post-intervention group, LoS reduction significantly reduced, with associated non-significant delirium and functional decline reduction
Roberts HJ et al. [13]	2022	Monocentric, retrospective pre- and post-co-management intervention	517 surgical patients (313 pre-intervention, 204 post-intervention)	Orthopedic Surgery (hip surgery)	Mortality, readmission rate, hospital-stay length, and surgical management time	Significant reduction in time from admission to surgical management and in LoS. The percentage of patients whose surgeries were performed under spinal anesthesia increased. No difference in 90-day readmission rate or mortality at 30 days, 90 days, or 1 year between groups.
Pollmann CT et al. [14]	2021	Single-center, prospective observational study	Usual care group: n = 94; orthogeriatric group: n = 103	Orthopedic Surgery	No delirium/SSD/delirium	Odds ratio for the development of SSD/delirium was lower in the orthogeriatric group
Bellas N et al. [15]	2020	Single-center, retrospective cohort study	491 surgical patients of whom 177 were comanaged	Orthopedic Surgery (hip surgery)	TTS, LoS, and postoperative complications	Majority of preoperative specialty consults did not meaningfully influence management and may have potentially increased morbidity by delaying surgery
Tadros RO et al. [16]	2015	Monocentric, retrospective pre- and post-co-management intervention	517 surgical patients (515 pre-intervention, 544 post-intervention)	Vascular Surgery	Mortality, patient safety, pain	Significant decrease in in-hospital mortality rates, patient safety, as measured by AHRQ, and improved pain scores. Resident surveys demonstrated perceived improvement in patient care and education
Tsunemitsu A et al. [17]	2024	Single-center retrospective cohort study	Conventional group: 332 patients; the co-management group: 418 patients	Orthopedic Surgery (hip surgery)		Co-management significantly reduced time to surgery and improved adherence to osteoporosis treatment and deep vein thrombosis prophylaxis guidelines, while length of stay, complications, and 30-day readmission rates did not differ significantly between groups.
Kim ES et al. [18]	2023	Single-center, retrospective cohort study	525 patients. passive surgical co-management group (n = 205), patients in the active SCM group (n = 320)	Urology	Clinical outcomes and perceptions of patients	Compared with passive SCM, active SCM was associated with a significantly shorter duration of co-management and trends toward shorter urology ward stays and fewer 30-day readmissions, with no differences in ICU transfers, in-hospital mortality, or inpatient care scores.
Fitzgerald SJ et al. [19]	2018	Monocentric, retrospective pre- and post-co-management intervention	Pre-co-management cohort: 1100 patients; co-management cohort: 1119 patients.	Orthopedic Surgery	LoS, ICU admissions, cases with complications, % mortality, 30-day readmission rate, and Hospital Consumer Assessment of Healthcare Providers and Systems scores	Statistically significant improvements in mean hospital LoS for total knee replacement, percentage of total knee replacement patients discharged home, and percentage of patients discharged home for primary total hip arthroplasty, complication rate, and 30-day readmission rate.
De Bueck U et al. [20]	2024	Monocentric, retrospective pre- and post-co-management intervention	59 patients “with” and 63 “without” geriatric co-management	Orthopedic Surgery (Native and Periprosthetic Joint Infections)	Delirium, pain, mobility, postoperative complications, and renal function	In the co-management group, delirium detection was higher, pain at discharge was lower, transfer ability improved more, and renal function was more frequently assessed, suggesting orthogeriatric co-management enhances recognition of medical issues in orthopedic patients.
Marchán-López Á et al. [21]	2024	Single-center, prospective cohort study	Pre co-management group: 227; co-management group: 475	Neurosurgery	Complications measured by the Accordion Severity Grading System, in-hospital mortality, and length of stay	Hospitalist co-management was associated with a reduced incidence of complications and length of stay in neurosurgical patients but there was no difference in in-hospital mortality.
Stier G et al. [22]	2018	Monocentric, retrospective pre- and post-co-management intervention	Pre co-management group: 163; co-management group: 261	Anesthesiology in surgical settings	Length of stay, complications	Significant reductions in length of stay (*p* < 0.05) were demonstrated for all surgical procedures. Significant reductions in complication rates and ileus observed
Dufour H et al. [23]	2020	Monocentric, prospective	229-patients	Neurosurgery	Hospital stay, complications	Reduction in the rates of complications, requests for specialist opinion, and hospital stay
Qato K et al. [24]	2022	Monocentric, pre- and post-co-management intervention	1062 patients, 520 pre-co-management, and 542 post-co-management	Vascular Surgery	Length of stay, complications, mortality	Decreased length of stay, re-admission, and mortality
Adogwa O et al. [25]	2017	Monocentric, retrospective	100 cases were retrospectively reviewed after initiation of the co-management protocol; 25 immediately preceding cases	Neurosurgery- lumbar spine surgery	Perioperative complications and clinical outcomes	In the co-managed cohort, the mean length of in-hospital stay and the mean duration of time between surgery and patient mobilization were shorter. In the co-managed cohort, the number of steps ambulated on the day of discharge was 2-fold higher
Gosch M et al. [26]	2016	Single-center, prospective cohort study	265 patients	Orthopedic Surgery (fragility fractures)	Mortality and functional outcome	One-year mortality was 29.4% in this cohort, significantly lower than in comparable trials.

**Table 2:** Distribution of surgical specialties across the included studies.

Specialty	n	%
Orthopedic surgery	8	36%
Vascular surgery	6	27%
Neurosurgery	4	18%
Oncologic surgery	3	14%
Urology	2	9%
ENT surgery	1	4.5%
Cardiac surgery	1	4.5%
Anesthesiology-led perioperative care	1	4.5%

## Data Availability

The original contributions presented in this study are included in the article. Further inquiries can be directed to the corresponding authors.

## Supplementary Materials

The following supporting information can be downloaded at: https://www.mdpi.com/article/10.3390/medicina62030579/s1, PRISMA 2020 Checklist. Reference [78] is cited in the supplementary materials.

## Abbreviations

The following abbreviations are used in this manuscript:

IMS	Internal Medicine Specialists
IM	Internal Medicine
ICU	Intensive Care Unit
LoS	Length of Hospital Stay
TTS	Time to Surgery
VTE	Venous Thromboembolism
MDA	Multidimensional Assessment
AHRQ	Agency for Healthcare Research and Quality
ENT	Ear–Nose–Throat Surgery
PHS	Perioperative Hospitalist Service
PSM	Post-Sternotomy Mediastinitis
SCM	Surgical Co-management
SSD	Subsyndromal Delirium

## References

[1] Tan Y.Y., Liaw F., Warner R., Myers S., Ghanem A. (2021). Enhanced Recovery Pathways for Flap-Based Reconstruction: Systematic Review and Meta-Analysis. Aesthetic Plast. Surg..

[2] De Vincentis A., Behr A.U., Bellelli G., Bravi M., Castaldo A., Cricelli C., Galluzzo L., Iolascon G., Maggi S., Martini E. (2020). Management of hip fracture in the older people: Rationale and design of the Italian consensus on the orthogeriatric co-management. Aging Clin. Exp. Res..

[3] Vitiello R., Bellieni A., Oliva M.S., Di Capua B., Fusco D., Careri S., Colloca G.F., Perisano C., Maccauro G., Lillo M. (2020). The importance of geriatric and surgical co- management of elderly in muscoloskeletal oncology: A literature review. Orthop. Rev..

[4] Van Grootven B., Mendelson D.A., Deschodt M. (2020). Impact of geriatric co-management programmes on outcomes in older surgical patients: Update of recent evidence. Curr. Opin. Anaesthesiol..

[5] Montero Ruiz E., Rebollar Merino Á., Rivera Rodríguez T., García Sánchez M., Agudo Alonso R., Barbero Allende J.M. (2015). Effect of comanagement with internal medicine on hospital stay of patients admitted to the Service of Otolaryngology. Acta Otorrinolaringol. Esp..

[6] Fernández-de-Velasco D., Villamor-Jiménez C., Carnero-Alcázar M., Sánchez-Del-Hoyo R., Pérez-Camargo D., Montero-Cruces L., Torres-Maestro B., Giraldo M.A., Reguillo-Lacruz F.J., Campelos-Fernández P. (2022). Co-Management Reduces Mortality in Post-Sternotomy Mediastinitis. Surg. Infect..

[7] Giannotti C., Massobrio A., Carmisciano L., Signori A., Napolitano A., Pertile D., Soriero D., Muzyka M., Tagliafico L., Casabella A. (2022). Effect of Geriatric Comanagement in Older Patients Undergoing Surgery for Gastrointestinal Cancer: A Retrospective, Before-and-After Study. J. Am. Med. Dir. Assoc..

[8] Shahrokni A., Tin A.L., Sarraf S., Alexander K., Sun S., Kim S.J., McMillan S., Yulico H., Amirnia F., Downey R.J. (2020). Association of Geriatric Comanagement and 90-Day Postoperative Mortality Among Patients Aged 75Years and Older With Cancer. JAMA Netw. Open.

[9] Thillainadesan J., Aitken S.J., Monaro S.R., Cullen J.S., Kerdic R., Hilmer S.N., Naganathan V. (2022). Geriatric Comanagement of Older Vascular Surgery Inpatients Reduces Hospital-Acquired Geriatric Syndromes. J. Am. Med. Dir. Assoc..

[10] Iberti C.T., Briones A., Gabriel E., Dunn A.S. (2016). Hospitalist-vascular surgery comanagement: Effects on complications and mortality. Hosp. Pract..

[11] McMillan S., Kim S.J., Tin A.L., Downey R.J., Vickers A.J., Korc-Grodzicki B., Shahrokni A. (2020). Association of frailty with 90-day postoperative mortality & geriatric comanagement among older adults with cancer. Eur. J. Surg. Oncol..

[12] Mudge A.M., McRae P., Donovan P.J., Reade M.C. (2020). Multidisciplinary quality improvement programme for older patients admitted to a vascular surgery ward. Intern. Med. J..

[13] Roberts H.J., Rogers S.E., Ward D.T., Kandemir U. (2022). Protocol-based interdisciplinary co-management for hip fracture care: 3 years of experience at an academic medical center. Arch. Orthop. Trauma. Surg..

[14] Pollmann C.T., Mellingsæter M.R., Neerland B.E., Straume-Næsheim T., Årøen A., Watne L.O. (2021). Orthogeriatric co-management reduces incidence of delirium in hip fracture patients. Osteoporos. Int..

[15] Bellas N., Stohler S., Staff I., Majk K., Lewis C., Davis S., Kumar M. (2020). Impact of Preoperative Specialty Consults on Hospitalist Comanagement of Hip Fracture Patients. J. Hosp. Med..

[16] Tadros R.O., Faries P.L., Malik R., Vouyouka A.G., Ting W., Dunn A., Marin M.L., Briones A. (2015). The effect of a hospitalist comanagement service on vascular surgery inpatients. J. Vasc. Surg..

[17] Tsunemitsu A., Tsutsumi T., Inokuma S., Imanaka Y. (2024). Effects of hospitalist co- management for hip fractures. J. Orthop. Sci..

[18] Kim E.S., Ohn J.H., Lim Y., Lee J., Kim H.W., Kim S.-W., Ryu J., Park H.-S., Cho J.H., Oh J.J. (2023). Effect of Active Surgical Co-Management by Medical Hospitalists in Urology Inpatient Care: A Retrospective Cohort Study. Yonsei Med. J..

[19] Fitzgerald S.J., Palmer T.C., Kraay M.J. (2018). Improved Perioperative Care of Elective Joint Replacement Patients: The Impact of an Orthopedic Perioperative Hospitalist. J. Arthroplast..

[20] De Bueck U., Kohlhof H., Wirtz D.C., Lukas A. (2024). Effects of an Integrated Geriatric-Orthopedic Co-management (InGerO) on the Treatment of Older Orthopedic Patients with Native and Periprosthetic Joint Infections. Z. Orthop. Unf..

[21] Marchán-López Á., Lora-Tamayo J., de la Calle C., Roldán L.J., Gómez L.M.M., de la Fuente I.S., Fernández M.C., Lagares A., Lumbreras C., Reyne A.G. (2024). Impact of a Hospitalist Co-Management Program on Medical Complications and Length of Stay in Neurosurgical Patients. Jt. Comm. J. Qual. Patient Saf..

[22] Stier G., Ramsingh D., Raval R., Shih G., Halverson B., Austin B., Soo J., Ruckle H., Martin R. (2018). Anesthesiologists as perioperative hospitalists and outcomes in patients undergoing major urologic surgery: A historical prospective, comparative effectiveness study. Perioper. Med..

[23] Dufour H., Rousseau-Ventos D. (2020). Optimizing medical postoperative care: Role of the hospitalist in a department of adult neurosurgery. Prospective comparativeobservational study. Neurochirurgie.

[24] Qato K., Ilyas N., Bahroloomi D., Conway A., Pamoukian V., Carroccio A., Giangola G. (2022). Hospitalist Co-Management of a Vascular Surgery Service Improves Quality Outcomes and Reduces Cost. Ann. Vasc. Surg..

[25] Adogwa O., Elsamadicy A.A., Vuong V.D., Moreno J., Cheng J., Karikari I.O., Bagley C.A. (2017). Geriatric comanagement reduces perioperative complications and shortens duration of hospital stay after lumbar spine surgery: A prospective single- institution experience. J. Neurosurg. Spine.

[26] Gosch M., Hoffmann-Weltin Y., Roth T., Blauth M., Nicholas J.A., Kammerlander C. (2016). Orthogeriatric co-management improves the outcome of long-term care residents with fragility fractures. Arch. Orthop. Trauma. Surg..

[27] Antonelli I.R., Gemma A., Capparella O. (2008). Orthogeriatric Unit: A thinking process and a working model. Aging Clin. Exp. Res.

[28] Pioli G., Giusti A., Barone A. (2008). Orthogeriatric care for thee lderly with hip fractures: Where are we?. Aging Clin. Exp. Res..

[29] Frondin C., Lunardelli M.L. (2010). Ortogeriatria un modello di assistenza ai pazienti anziani con frattura di femore. Ital. J. Med..

[30] Aw D., Sahota O. (2014). Orthogeriatrics Moving Forward Age and Ageing. Age Ageing.

[31] Shenning K., Deiner G. (2015). Post operative delirium in geriatric patient. Anestesiol. Clin..

[32] Kazmierski J., Kowman M., Banach M., Fendler W., Okonski P., Banys A., Jaszewski R., Rysz J., Sobow T., Kloszewska I. (2010). The use of DSM-IV and ICD-10 criteria and diagnostic scales for delirium among cardiac surgery patients: Results from the IPDACS study. J. Neuropsychiatry Clin. Neurosci..

[33] van der Mast R.C., Roest F.H. (1996). Delirium after cardiac surgery: A critical review. J. Psychosom. Res..

[34] Taxis J., Spoerl S., Broszio A., Eichberger J., Grau E., Schuderer J., Ludwig N., Gottsauner M., Spanier G., Bundscherer A. (2022). Postoperative Delirium after Reconstructive Surgery in the Head and Neck Region. J. Clin. Med..

[35] Ishibashi-Kanno N., Takaoka S., Nagai H. (2020). Postoperative delirium after reconstructive surgery for oral tumor: A retrospective clinical study. Int. J. Oral Maxillofac. Surg..

[36] Kuwahara M., Yurugi S., Mashiba K., Iioka H., Niitsuma K., Noda T. (2008). Postoperative delirium in plastic or dermatologic surgery. Eur. J. Plast. Surg..

[37] American Psychiatric Association (2013). Diagnostic and Statistical Manual of Mental Disorders.

[38] Ely E.W., Margolin R., Francis J., May L.R., Truman B.R., Dittus R., Speroff T., Gautam S., Bernard G.R., Inouye S.K. (2001). Evaluation of delirium in critically ill patients: Validation of the Confusion Assessment Method for the Intensive Care Unit (CAM- ICU). Crit. Care Med..

[39] Albert M.S., Levkoff S.E., Reilly C., Liptzin B., Pilgrim D., Cleary P.D., Evans D., Rowe J.W. (1992). The delirium symptom interview: An interview for the detection of delirium symptoms in hospitalized patients. J. Geriatr. Psychiatry Neurol..

[40] Maldonado J.R. (2013). Neuropathogenesis of delirium: Review of current etiologic theories and common pathways. Am. J. Geriatr. Psychiatry.

[41] Cerejeira J., Firmino H., Vaz-Serra A., Mukaetova-Ladinska E.B. (2010). The neuroin-flammatory hypothesis of delirium. Acta Neuropathol..

[42] DeVito R.G., Craft L., Campbell C.A., Stranix J.T. (2023). Optimizing perioperative outcomes in autologous breast reconstruction. Gland Surg..

[43] Desborough J.P. (2000). The stress response to trauma and surgery. Br. J. Anaesth..

[44] Young L.H., Wackers F.J.T., Chyun D.A., Davey J.A., Barrett E.J., Taillefer R., Heller G.V., Iskandrian A.E., Wittlin S.D., Filipchuk N. (2009). DIAD Investigators. Cardiac outcomes after screening for asymptomatic coronary artery disease in patients with type 2 diabetes: The DIAD study: A randomized controlled trial. JAMA.

[45] Douketis J.D., Spyropoulos A.C., Spencer F.A., Mayr M., Jaffer A.K., Eckman M.H., Dunn A.S., Kunz R. (2012). Perioperative management of antithrombotic therapy: Antithrombotic Therapy and Prevention of Thrombosis, 9th ed: American College of Chest Physicians Evidence-Based Clinical Practice Guidelines. Chest.

[46] Dhatariya K., Levy N., Kilvert A., Watson B., Cousins D., Flanagan D., Hilton L., Jairam C., Leyden K., Lipp A. (2012). NHS Diabetes guideline for the perioperative management of the adult patient with diabetes. Diabet. Med..

[47] Barker P., Creasey P.E., Dhatariya K., Levy N., Lipp A., Nathanson M.H., Penfold N., Watson B., Woodcock T. (2015). Peri-operative management of the surgical patient with diabetes. Anaesthesia.

[48] Kjærgaard K., Wheler J., Dihge L., Christiansen P., Borgquist S., Cronin-Fenton D. (2024). Impact of type 2 diabetes on complications after primary breast cancer surgery: Danish population-based cohort study. Br. J. Surg..

[49] Braga M., Ljungqvist O., Soeters P., Fearon K., Weimann A., Bozzetti F. (2009). ESPEN Guidelines on parenteral nutrition: Surgery. Clin. Nutr..

[50] Clark A., Imran J., Madni T., Wolf S.E. (2017). Nutrition and metabolism in burn patients. Burn. Trauma..

[51] Fiser C., Crystal J.S., Tevis S.E., Kesmodel S., E Rojas K. (2021). Treatment and Survivorship Interventions to Prevent Poor Body Image Outcomes in Breast Cancer Survivors. Breast Cancer.

[52] Yang A., Sokolof J., Gulati A. (2018). The effect of preoperative exercise on upper extremity recovery following breast cancer surgery: A systematic review. Int. J. Rehabil. Res..

[53] Toohey K., Hunter M., McKinnon K., Casey T., Turner M., Taylor S., Paterson C. (2023). A systematic review of multimodal prehabilitation in breast cancer. Breast Cancer Res. Treat..

[54] Doganay E., Moorthy K. (2019). Prehabilitation for esophagectomy. J. Thorac. Dis..

[55] Hijazi Y., Gondal U., Aziz O. (2017). A systematic review of prehabilitation programs in abdominal cancer surgery. Int. J. Surg..

[56] Loewen I., Jeffery C.C., Rieger J., Constantinescu G. (2021). Prehabilitation in head and neck cancer patients: A literature review. J. Otolaryngol. Head Neck Surg..

[57] Schneider S., Armbrust R., Spies C., du Bois A., Sehouli J. (2020). Prehabilitation programs and ERAS protocols in gynecological oncology: A comprehensive review. Arch. Gynecol. Obstet..

[58] Sykes K.J., Gibbs H., Farrokhian N., Arthur A., Flynn J., Shnayder Y., Kakarala K., Nallani R., Smith J.B., Penn J. (2023). Pilot randomized, controlled, preoperative intervention for nutrition trial in head and neck cancer. Head Neck.

[59] Palackic A., Suman O.E., Porter C., Murton A.J., Crandall C.G., Rivas E. (2021). Rehabilitative Exercise Training for Burn Injury. Sports Med..

[60] de Sire A., Losco L., Lippi L., Spadoni D., Kaciulyte J., Sert G., Ciamarra P., Marcasciano M., Cuomo R., Bolletta A. (2022). Surgical Treatment and Rehabilitation Strategies for Upper and Lower Extremity Lymphedema: A Comprehensive Review. Medicina.

[61] Gianesini S., Tessari M., Bacciglieri P., Malagoni A.M., Menegatti E., Occhionorelli S., Basaglia N., Zamboni P. (2017). A specifically designed aquatic exercise protocol to reduce chronic lower limb edema. Phlebology.

[62] Szolnoky G., Tuczai M., Macdonald J.M., Dosa-Racz E., Barsony K., Balogh M., Szabad G., Dobozy A., Kemeny L. (2018). Adjunctive role of manual lymph drainage in the healing of venous ulcers: A comparative pilot study. Lymphology.

[63] Brix B., Apich G., Rössler A., Walbrodt S., Goswami N. (2021). Effects of physical therapy on hyaluronan clearance and volume regulating hormones in lower limb lymphedema patients: A pilot study. Sci. Prog..

[64] Fernández-Guarino M., Bacci S., Pérez González L.A., Bermejo-Martínez M., Cecilia-Matilla A., Hernández-Bule M.L. (2023). The Role of Physical Therapies in Wound Healing and Assisted Scarring. Int. J. Mol. Sci..

[65] Finnerty C.C., Jeschke M.G., Branski L.K., Barret J.P., Dziewulski P., Herndon D.N. (2016). Hypertrophic scarring: The greatest unmet challenge after burn injury. Lancet.

[66] Auerbach A.D., Watcher R., Cheng Q., Maselli J., McDermott M., Vittinghoff E., Berger M.S. (2010). Comanagement of Surgical Patients Between Neurosurgeons and Hospitalists. Arch. Intern. Med..

[67] Levin D., Glasheen J.J. (2011). Achieving Comanagement’s Potential Requires System Redesign and Hospitalist-Focused Training. Arch. Intern. Med..

[68] Cheng H.Q. (2015). Co-management Hospitalist Services for Neurosurgery. Neurosurg. Clin. N. Am..

[69] O’Malley P. (2010). Surgical Comanagement: Can We Afford To Do This?. Arch. Intern. Med..

[70] Watcher B. (2010). Hospitalist Co-Management of Neurosurgery Patients: The Inside Story of a Winning Intervention. Watcher’s World.

[71] Watcher R.M. (2014). Hospitalist Workload. The Search for the Magic Number. JAMA Intern. Med..

[72] Gesensway D. (2010). Neurosurgery: The last comanagement frontier. Today’s Hospitalist.

[73] Genther D., Gourin C.G. (2015). Effect of comorbidity on short- term outcomes and cost of care after head and neck cancer surgery in the elderly. Head Neck.

[74] Makary M.A., Segev D.L., Pronovost P.J., Syin D., Bandeen-Roche K., Patel P., Takenaga R., Devgan L., Holzmueller C.G., Tian J. (2010). Frailty as a predictor of surgical outcomes in older patients. J. Am. Coll. Surg..

[75] Turner G., Clegg A. (2014). Best practice guidelines for the management of frailty: A British Geriatrics Society, Age UK and Royal College of General Practitioners report. Age Ageing.

[76] Stuck A.J., Hegger M., Hammer A., Minder C.E., Beck J.C. (2002). Home visits to pre-vent nursing home admissions and functional decline in elderly people: Systematic review and meta-regression analysis. J. Amer Med. Assoc..

[77] Pilotto A., Ferrucci L., Franceschi M., D’AMbrosio L.P., Scarcelli C., Cascavilla L., Paris F., Placentino G., Seripa D., Dallapiccola B. (2008). Development and validation of a multidimensional prognostic index for one-year mortality from comprehensive geriatric assessment in hospitalized older patients. Rejuvenation Res..

[78] Page M.J., McKenzie J.E., Bossuyt P.M., Boutron I., Hoffmann T.C., Mulrow C.D., Shamseer L., Tetzlaff J.M., Akl E.A., Brennan S.E. (2021). The PRISMA 2020 statement: An updated guideline for reporting systematic reviews. BMJ.

